# Design of a Care Pathway for Preventive Blood Pressure Monitoring: Qualitative Study

**DOI:** 10.2196/13048

**Published:** 2019-05-03

**Authors:** Carlijn Geerse, Cher van Slobbe, Edda van Triet, Lianne Simonse

**Affiliations:** 1 Product Innovation Management Department Faculty of Industrial Design Engineering Delft University of Technology Delft Netherlands

**Keywords:** eHealth, blood pressure monitoring, at-risk patients, secondary preventive care, care pathway, design

## Abstract

**Background:**

Electronic health (eHealth) services could provide a solution for monitoring the blood pressure of at-risk patients while also decreasing expensive doctor visits. However, a major barrier to their implementation is the lack of integration into organizations.

**Objective:**

Our aim was to design a Care Pathway for monitoring the blood pressure of at-risk patients, in order to increase eHealth implementation in secondary preventive care.

**Methods:**

A qualitative design study was used in this research. Data were collected by conducting visual mapping sessions including semistructured interviews with hypertension patients and doctors. The data were transcribed and coded and thereafter mapped into a Care Pathway.

**Results:**

Four themes emerged from the results: (1) the current approach to blood pressure measuring has disadvantages, (2) risk and lifestyle factors of blood pressure measuring need to be considered, (3) there are certain influences of the at-home context on measuring blood pressure, and (4) new touchpoints between patients and health professionals need to be designed. These in-depth insights combined with the visualization of the current blood pressure process resulted in our Care Pathway design for monitoring the blood pressure of at-risk patients as secondary preventive care.

**Conclusions:**

The Care Pathway guides the implementation of eHealth devices for blood pressure self-measurement. It showcases the pathway of at-risk patients and increases their involvement in managing their blood pressure. It serves as a basis for a new service using eHealth.

## Introduction

### Electronic Health and Relations Between Patient and Health Professional

The arrival of internet-enabled health (electronic health, eHealth), which ushered in large online health databases and platforms, caused a change in the attitude of health-conscious consumers and their relationship with health professionals. Health professionals are no longer the main source of health care information. Many consumers seek confirmation of their general practitioners’ (GP) decisions on the Internet, enabling them to be more active in their health management [1-3]. For instance, in 2014, 73% of Dutch citizens obtained health-related information online [4]. The self-efficacy of patients has increased and the interaction between patient and GP has become more patient-centered [1]. The perception that patients should not be bothered with too much detailed information because they are unable to cope has become outdated [5]. According to the World Health Organization, patient participation is the new paradigm in the global diffusion of eHealth—the use of information and communication technologies (ICT) for health. It enables increased self-managed care [6] and patient-centered care [7]. Self-management services can help patients understand their condition better and adjust their decision making accordingly [8]. They value data and information exchange more and want to be fully informed and continuously involved in the decision making in their treatment [3,9]. The overall challenge is how to embed these changes in new models of care—models that enable participation in the decision making enabled by eHealth and that share health care data, information, and insights into the relationship between patients and health professionals.

### Integration Barriers for eHealth

To deal with higher demand for health care, social care, and social pensions from an ageing population and in the face of a shrinking workforce, the care models must be redesigned. Costs must be reduced without compromising the quality and accessibility of health care [10]. To achieve value-based health coverage, preventive eHealth services such as tracking diseases and monitoring public health are a necessity [11]. eHealth enabling early detection of diseases has a high positive impact on quality and costs. When symptoms are detected in an early stage, the likelihood of chronic diseases is reduced and high costs can be avoided [12]. Moreover, health monitoring as part of eHealth services could make it possible to avoid expensive hospital visits, and certain treatments of patients at risk can be done at home [13]. To date, however, there have been few successful implementations of eHealth services [14,15]. The slow adoption of services by users [16], the lack of collaboration between health care organizations and their systems [17], a shortage of funding [13], and the lack of care models to guide implementation are factors that obstruct the development and implementation of sustainable eHealth services [18]. This research therefore focuses on the design of a care model for eHealth services. We chose to study the monitoring of blood pressure, as it is an indicator for multiple high-cost diseases [19].

### Blood Pressure Monitoring With eHealth for At-Risk Patients

Self-measured blood pressure devices have been around for many years but have not been extensively implemented in health services [20]. Early on, health professionals considered the results of these devices unreliable and most patients found them difficult to use [3]. Over the years, these eHealth products have improved considerably and are now trustworthy. A self-measured blood pressure device consists of an armband with sensors that measure one’s blood pressure and heart rate and a monitor that presents the results and automatically transfers the data to an online app. This Web service provides the patient with data history and tracking overviews. For people with a higher risk or first symptoms of health problems, the use of self-measurement services could help detect illness or complications at an earlier stage. We refer to this group as at-risk patients. In the current health care system, the patient’s blood pressure is measured during a GP consultation or at home. This is a reactive care service with one moment of measurement that does not provide constant tracking and feedback on one’s blood pressure. Researchers have found in favor of at-home measurements because it is difficult to take accurate blood pressure measurements in an unnatural environment [21]. The Dutch health care system organizes eHealth blood pressure monitoring with a preventive focus under a reimbursement-based financial model, in which disease complications and impairment are detected at an early stage, the so-called tertiary level of care provision [22]. The use of this model for eHealth services could shift towards secondary preventive care at the GP’s office by helping detect complications in at-risk patients. eHealth can have major cost effects in avoiding a shift to primary care by specialists at the hospital. Regular blood pressure measuring, and thus earlier detection of hypertension, could reduce the likelihood of (exacerbation of) chronic diseases such as kidney failure [23]. A recent systematic review found that self-management of hypertension facilitated an increase in health, patient knowledge and involvement, greater cost-effectiveness, and a more accurate reading of results because the patient is in a natural environment [24]. The high blood pressure of this group of patients cannot be cured, but it can be managed. The estimated cost effects for the Netherlands are high, with 1.7 million people with chronic kidney disease, a number that is rising by 2000 per year [25]. Monitoring with eHealth could be a solution for monitoring patients’ blood pressure while also decreasing expensive specialist visits.

### Care Pathway Design to Overcome Barriers

The current approach to monitoring blood pressure of at-risk patients is used as tertiary preventive care, while eHealth services can make the shift to secondary preventive care. In order to make this shift, the current situation of monitoring blood pressure was researched. Communication and data streams between all the actors are important to design a Care Pathway for secondary preventive care. The major barrier that must be overcome in implementing preventive eHealth services is its lack of integration into organizations. The embedding of ICT requires care provider pathways that cross organizational boundaries. Care Pathways can be described as a concept for making patient-centered care operational [26] and gaining insight into how an organization can improve its services [27]. According to the European Pathway Association “the objective of a Care Pathway is to enhance the quality of care by improving patient outcomes, promoting patient safety, increasing patient satisfaction, and optimizing the use of resources” [28]. Studies have shown that Care Pathways improved communication between professionals [29] and clarified the division of roles and responsibilities [30]. With these qualities, a Care Pathway could help overcome some of the implementation barriers for preventive eHealth services. Therefore, in this paper, we concentrated on how to create a Care Pathway.

### Research Question

The objective of our research is to design a Care Pathway for monitoring blood pressure of at-risk patients, focusing on the exchange of data and communication between the individuals involved. To do so, we will first define the current situation and use this to create a Care Pathway that focuses on clarifying how processes should be carried out and by whom in order to increase the adoption of eHealth services [31]. The following research question was formulated: How can a Care Pathway to monitor blood pressure of “at-risk patients” be designed in order to increase the implementation of eHealth in secondary preventive care?

## Methods

### Study Design

For this research, a qualitative approach is used to explore this relatively new field. A qualitative research approach allows us to gain a better understanding of underlying opinions, reasons, and emotions [32]. A phenomenon study was chosen in order to unravel the participants’ real experiences and understanding of the blood pressure monitoring service. According to Suter [33], the focus in a phenomenon study is on the essence of an experience, that is, on trying to understand the basic structure of that experience and interpreting the meaning it has for a person or group.

### Data Collection

A visual mapping toolkit with an interview guide were used to organize generative sessions with the participants. As a result, a Care Pathway was co-created [34]. This was done to gain a better understanding of the data streams between the different individuals and locations where blood pressure is monitored. Three types of qualitative research data were obtained: audio recordings of the interviews, documentation of institutes, and visuals created in the visual mapping sessions.

#### Sampling

Purposeful sampling was used to identify and select individuals who had experiences with the studied phenomenon of eHealth [35]. For the design of a Care Pathway, it is especially important to involve different stakeholders in the development phase of the pathway [31]. In this research, we selected people diagnosed with high blood pressure and researched their current health management and their interaction with health professionals. Seven interviews were conducted in total: one specialist, three patients, and three GPs. An overview of the participants can be found in Table 1.

All interviewed patients had been diagnosed with high blood pressure after several measurements taken by their GP and 24-hour measurement at home. For one day they carried a blood pressure device around that measured their blood pressure every 30 minutes for 24 hours. All were prescribed medicines and had a GP consultation at least once a year. Patient 1 is part of an eHealth monitoring program and therefore regularly measures his blood pressure by himself. Patients 2 and 3 obtain information about their blood pressure only during their yearly consultation.

#### Ethics

The research was performed according to the principles of the Helsinki Declaration and Nuremberg and was checked and reviewed by the human research ethics committee (HREC) of the Delft University of technology [36]. We did not involve vulnerable groups of children or patients over age 65 in our sample design. The participation in our research was voluntary. The methods and data used were checked and approved by the HREC and the data steward of the Industrial Design Engineering faculty.

#### Visual Mapping Session

**Figure 1:**
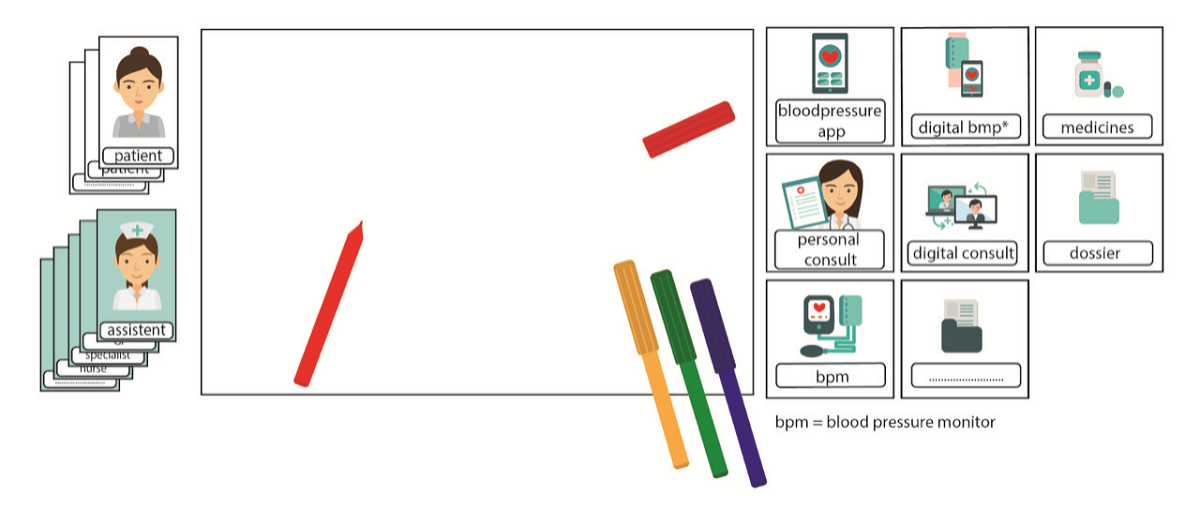
Visual mapping toolkit.

To generate insights into the interactions between the actors and the data streams, a visual mapping toolkit was designed, as introduced and outlined in a design research protocol by Meeuwen, Walt Meijer, and Simonse [18]. This toolkit consisted of two-dimensional representations of different people and products involved in the medical process, pencils, markers and blank paper. An overview of the design toolkit can be found in Figure 1. A pilot session with test participants was done to improve the toolkit before conducting the sessions with the participants in this sample.

**Table 1:** Sample of participants.

Participants		Gender	Situation	Health service experience	eHealth – blood pressure experience
**Patients**					
	Patient 1	Male	Kidney disease	±20 years	Yes
	Patient 2	Female	Pregnancy hypertension	±20 years	No
	Patient 3	Male	Stress due to work	±2 years	No
**General practitioners (GPs)**					
	GP 1	Male	Working as GP in own practice	±40 years	No
	GP 2	Male	Working as medical advisor at a health insurance company	±20 years	Yes
	GP 3	Male	Working as GP, community practice (formerly a trauma specialist)	±3 years	No
**Specialist**					
	Specialist 1	Male	Specialist Internal medicine	±11 years	Yes

Six mapping sessions were performed with three patients and three GPs. The visual mapping session was built up in several levels since people’s needs and values are often difficult to discuss [37]. The participants were first asked to visualize the current situation of blood pressure measuring and monitoring, and then to visualize a pathway for blood pressure monitoring at home. During the session, each participant was asked to reflect on their own needs and values [37], since they are the expert on their own experience area, including their interactions with the eHealth device and the ICT infrastructure [34]. The facilitator of the session played a guiding role during the visual mapping sessions and led the layering questioning. The visual mapping results were photographed at the end of each session in order to compare all the results.

#### Semistructured Interviews

The interview guide was constructed for the different actors, providing structure to the interviews while still maintaining the freedom to go into depth on certain topics [38]. The structure helped in analyzing the obtained data and finding patterns in it. All the interviews were audio recorded and transcribed for descriptive validity [39].

### Data Analysis

Triangulation was used in the analysis by clustering the data from in-depth interviews, visual mapping sessions, and documentation [40]. First, initial coding identified important (groups of) words in the data, and these were labeled accordingly [41]. Second, with focused coding, codes were clustered and categorized to make a code book. Finally, multiple coding was used to avoid subjectivity in interpretation [42]. Subjectivity in interpretation was partly avoided by making use of investigator triangulation [40]. The outcomes of the mapping sessions were visually analyzed by comparing the different pathways of the participants. The similarities in the pathways have been considered and have led to the design of the current blood pressure monitoring situation and the new Care Pathway. The data analysis of the interviews served as a basis and check for the visual Care Pathway.

## Results

### Current Blood Pressure Monitoring Process

**Figure 2:**
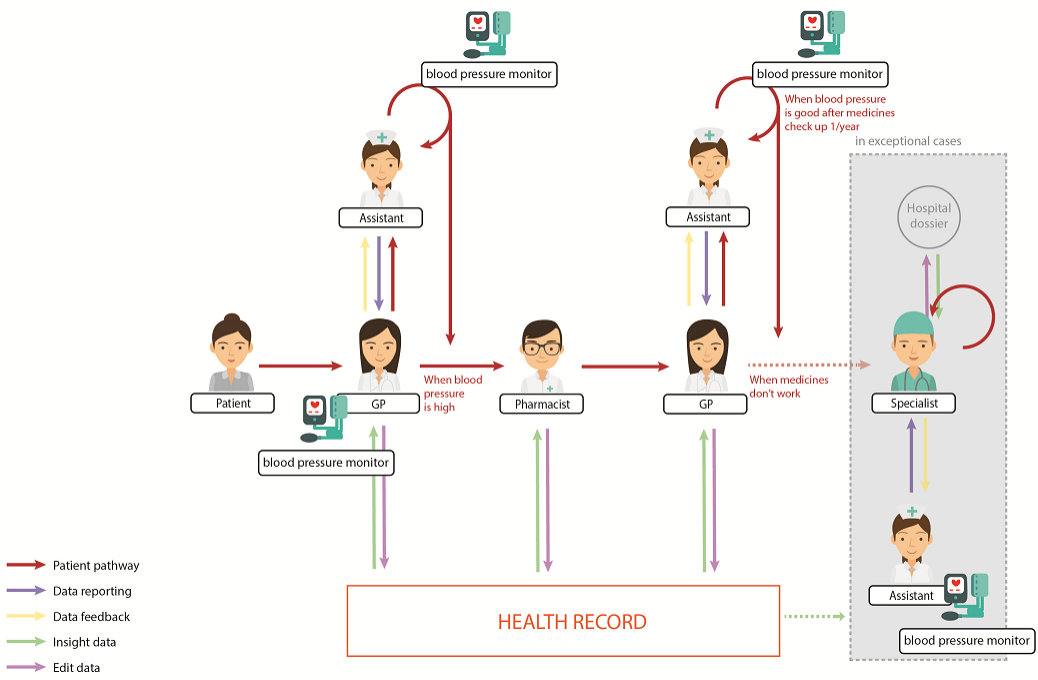
Current blood pressure process. GP: general practitioner.

Before designing the Care Pathway, the current blood pressure process was visualized (Figure 2), drawn from the data obtained in the visual mapping sessions including the explanations in the interviews. In the current situation, the at-risk patient’s blood pressure is monitored during a GP consultation as a physical check. In most cases, the GP assistant measures the patient’s blood pressure and hands the results to the GP, who enters all the measured data manually into a health record system. If the patient’s blood pressure is too high, a new appointment is made to repeat the measurement. If the blood pressure remains too high after several measurements have been taken within 3 months, the GP will prescribe medicines to lower and control the blood pressure. The pharmacist modifies the prescription if necessary. In exceptional cases, or when medicines do not lower the blood pressure, the patient will be referred to a specialist, who measures the blood pressure again.

**Figure 3:**
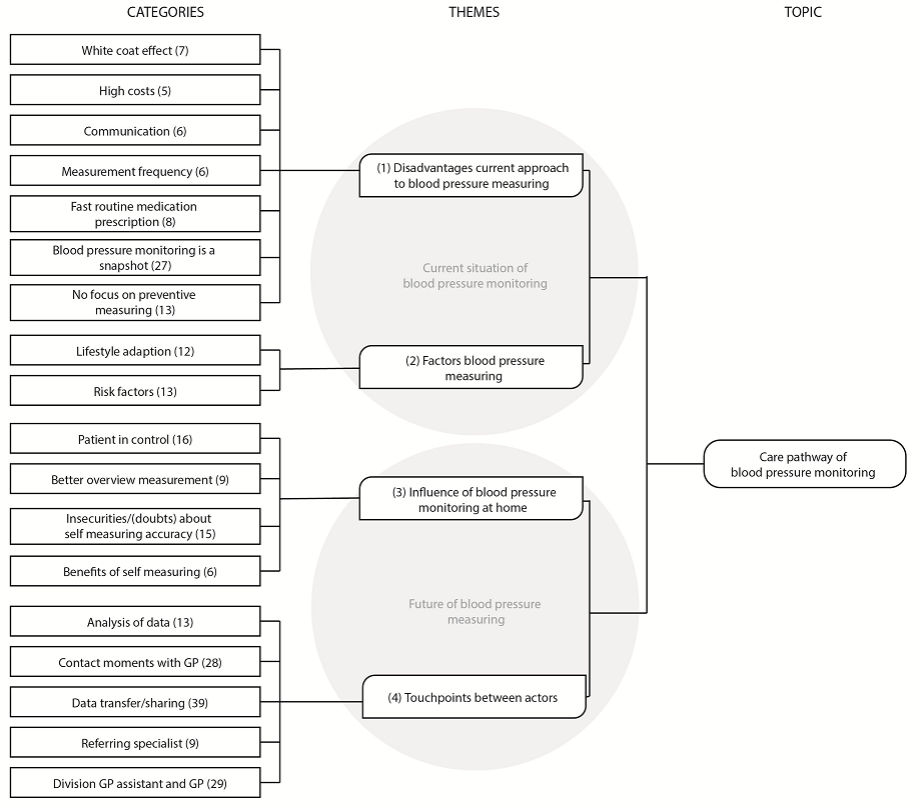
Coding tree with the analysis results of codes (number in brackets), categories and themes. GP: general practitioner.

On four themes that emerged from the data analysis, there is common agreement: (1) the current approach to blood pressure measuring has disadvantages, (2) risk and lifestyle factors of blood pressure measuring need to be taken into account, (3) there are certain influences of the at-home context on measuring blood pressure, and (4) new touchpoints between key actors need to be designed. Themes 1 and 2 relate to the current situation of blood pressure measuring and point out strengths and pitfalls. Theme 3 reveals important insights into self-measuring and monitoring blood pressure at home, and the perceived positive effects on patients’ well-being, accuracy of measurement results, and health care costs and the negative effect of insecurity. Theme 4 extracts the new elements in the co-designed Care Pathway. The coding tree in Figure 3 shows the four themes with underlying categories and codes. All insights on these themes were considered in the design of the Care Pathway for blood pressure monitoring.

### Theme 1: Disadvantages of Current Approach to Blood Pressure Measuring

One of the essential disadvantages of blood pressure measuring is that it is a snapshot of one measurement that varies over time. This drawback was quoted 27 times by participants (see the number 27 in brackets in Figure 2). Hereafter, we will refer in the text to the number of quotes as (# quotes). With the current approach to blood pressure measuring, there are little data about people’s blood pressure, as it is measured only a few times a year during the GP consultation. Additionally, these measurements are not always accurate due to the “white coat effect” (7 quotes), meaning that some people become stressed when their blood pressure is being measured by a GP, resulting in an unrealistically high blood pressure. This effect can be reduced by taking multiple measurements over a certain period or by measuring in a different environment:

When I find a very high blood pressure, I just let the patients sit and relax and will do another measurement again. But you would prefer to have some more measurements over time.

GP 2

The GP has a prominent role in analyzing the results and deciding on the medication. In most cases the patient is not given insight into the blood pressure results. When the blood pressure of a patient is too high after several measurements, the GP often decides to switch to medication:

We usually treat at-risk patients with medicine. If one medicine does not work, you try a second one, then a third and maybe a fourth, all with an increasing dose.

GP 1

Furthermore, our participants validated that the current system is curative rather than preventive:

I do know that a lot of medication is prescribed, which may not be necessary. I am terrified how many medicines people swallow every day.

Patient 2

Participants suggested that this could be improved with the Care Pathway for blood pressure monitoring.

### Theme 2: Risk and Lifestyle Factors on Blood Pressure Measuring

According to the GPs, blood pressure is considered a small piece of the complete health picture. Factors like smoking, diabetes, and high cholesterol in combination with blood pressure are important risk factors for heart and vascular diseases (13 quotes). Those risk factors are part of someone’s lifestyle. The patients often stated that they have tried to adopt a healthier lifestyle after being diagnosed with high blood pressure (12 quotes).

The GP prescribed medicines but I didn’t want to take them. So, I started doing sports andPatient 3losing weight, but getting stress under control was hard.

Patient 3

Both the specialist internal medicine and patients pointed out that lifestyle is hard to change, while lifestyle modification could also help lower blood pressure.

### Theme 3: Influence of the At-Home Context on Blood Pressure Measuring

Measuring blood pressure at home enables patients to be in control of their own health care (16 quotes). Moreover, it gives a better overview and therefore more accurate measurements (9 quotes). Patients measure their blood pressure in a trusted environment at standard moments. This increases the reliability of the measurements. The benefit of self-measuring is that it reduces the influence of different context factors that can affect a person’s blood pressure temporarily (6 quotes). Patients can decide when to measure and analyze their measurements themselves:

People enjoy measuring their blood pressure at home because it gives them power over their own health and they can try to influence it by looking at their lifestyle: if I eat less salt, does my blood pressure drop?

GP 1

We also observed a difference in attitude among the participants towards blood pressure measuring. GP 1 and Patients 2 and 3 mentioned that this might put too much emphasis on their condition and that they do not want to be confronted with it too much:

Blood pressure measuring is a hot item, but it can make people feel unhappy as well. We should not exaggerate it, but it’s definitely an important factor.

GP 1

Patients pointed out that measuring their blood pressure at home has benefits (6 quotes), but they do have insecurities about the self-measurement (15 quotes). Patients are insecure about how to perform a measurement and self-interpret the results, and they are anxious about the accuracy of the product:

Every time I measure, I wonder: do I measure technically good? Do I operate the product in the right way? Do I measure at the right moment?

Patient 1

### Theme 4: New Touchpoints Between Patients and Doctors

To ensure the successful implementation of the eHealth product, new touchpoints between the different key actors were defined. Both the patients and the GPs want to keep scheduling regular consulting moments, which can be with the GP or the GP assistant. From the analysis of all co-creation sessions, we concluded that patients prefer to measure their own blood pressure at home but would also like to have contact with their GP occasionally. All patients mentioned that they would prefer *data exchange* of the results through an app (39 quotes) or email and would prefer occasional feedback on the data overviews from a care professional, either the GP or GP assistant (29 quotes). The GPs are also in agreement that there should be personal contact between a GP and patient because:

...you can use personal contact to motivate patients.

GP 2

The patients want to be able to view and analyze their own data to have a sense of control (13 quotes):

I like the feeling that I can look at my own results. That is important to me … I want to keep an eye on my GP.

Patient 1

Concerning the frequency of contact moments, interestingly the patient with eHealth experience wanted to be checked more frequently and preferred having this done by the GP:

...a GP would have probably responded to that, but the assistant did not.

Patient 1

The others agreed that the current frequency of GP consultations, every 6 or 12 months, is preferable (28 quotes), as they are not then constantly reminded about their blood pressure. There is a further division in whom patients want to share and discuss their data with. Patients 2 and 3 were satisfied with having a consultation once a year done by a GP assistant, under the supervision of the GP, who has greater expertise. The participants also wanted to keep the specialist in the loop to help with the interpretation of the blood pressure data concerning the risks of their particular health condition (9 quotes). Overall, we concluded that participants would prefer a Care Pathway that includes both self-measurement and professional care monitoring.

### Integrated Care Pathway Design

**Figure 4:**
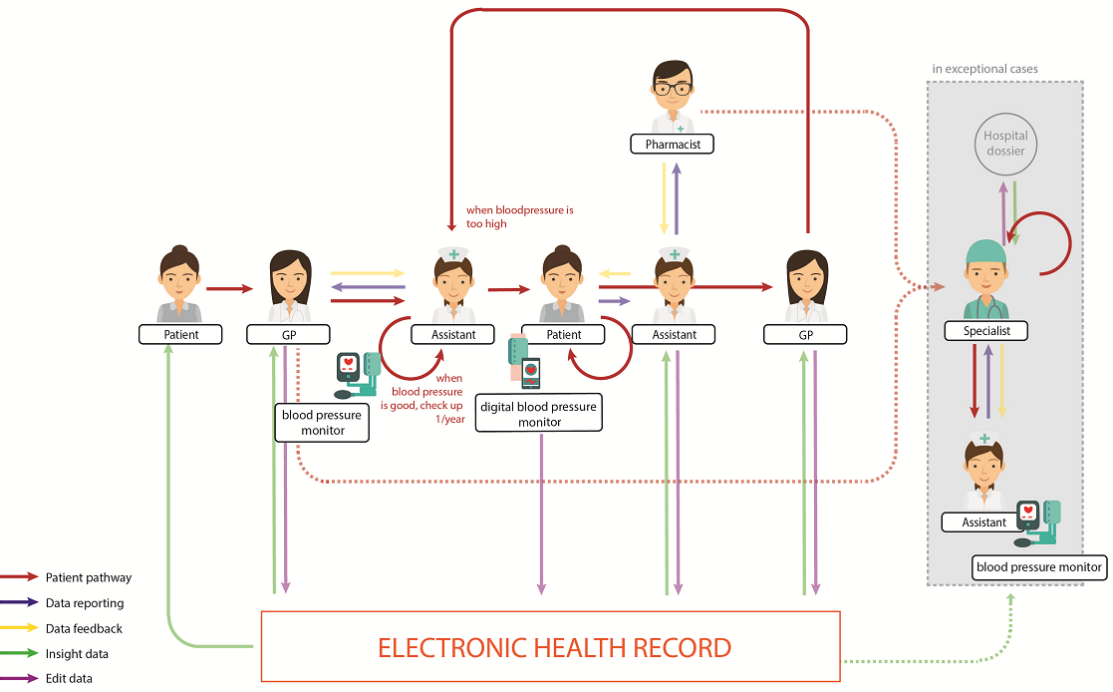
Care pathway of blood pressure monitoring for preventive care.

The new design of the Care Pathway for at-risk patients as secondary preventive care is displayed in Figure 4. As in the current situation, the patient visits the GP, and their blood pressure is measured by the GP assistant. The design of the pathway differs in that it provides a new secondary preventive health care service:

- When the blood pressure is too high, the patient will receive a blood pressure monitor for at-home use and start to self-measure the blood pressure, generating an overview over a period of 3 months.
- The measured data will be saved by the device and automatically transferred to the health record system.
- The GP assistant receives an e-message if the patient’s blood pressure is too high.
- Instantly the GP assistant analyzes the data and provides direct feedback to the patient.
- In some incidents, this feedback message might state that the data needs to be checked by the GP.
- For most incidents, the GP assistant is expected to do the monitoring and communicates with the patient.
- In stable situations, there is a (digital) contact moment every 3 months.
- Furthermore, the GP assistant will also communicate with the pharmacists on changes in medication doses.
- The blood and medication monitoring remain under the supervision of the GP, who will make the final decisions concerning medication and further diagnoses and treatments.
- Another aspect that is similar to the current process is that if the GP cannot control the blood pressure or if a patient has many other risk factors, the GP will refer the patient to a specialist (in most cases the internist). The specialist will examine the patient further.

## Discussion

### Principal Results

This study developed a Care Pathway with a new secondary preventive care service that embeds at-home blood pressure measurement activities into the health care organization of diagnoses and treatment of at-risk patients. The study showed that in the current situation the GP measures the blood pressure during a consultation and prescribes medicines when the blood pressure is too high after a couple of measurements. This routine activity for at-risk patients is considered to belong to tertiary preventive care, where the care for patients is curative and reimbursed against higher costs. This Care Pathway design realizes a service shift to secondary preventive care, in which incidents and additional diseases can be tracked down in an earlier stage.

First, the routine activity is performed at the source, the patient is self-involved in executing the blood pressure measurements, leading to a feeling of being in control but also to a commonly acknowledged need to exchange and share the data with professional care providers. Second, this new touchpoint of data sharing and professional monitoring is performed by GP assistants with the additional benefit of earlier detection and reinforcement of lifestyle changes. Third, the data reporting is automated from the eHealth device into the health record system, integrating the patient as an implicit user of the health record system. A principal aspect of the design of a Care Pathway is the gathering of in-depth insights, not only on the current process but also on the future of health care services envisioned in co-creation sessions.

### Comparison With Prior Work and Theoretical Implications

With this pathway design study, we showed that the activity of blood pressure monitoring can be shifted to the patient who can use an eHealth device at home under the precondition that the use is embedded into the Care Pathway, with a point of contact and safety service of incident monitoring at the organization of the GP practice.

These in-depth findings confirm the principal drive behind the patient-centered movement in which patients are actively involved and take greater responsibility for their own health [2]. This pathway design incorporates an eHealth blood pressure device that gives patients immediate feedback and insights into their health data, providing a direct impulse to improve their health outcomes. Patients can first try to manage or reduce their blood pressure themselves, instead of relying immediately on medication prescriptions.

Reasons to measure blood pressure at home have been researched in other studies [20,43]. An initial benefit of measurement at home has already been confirmed by previous research that found lower measurement outcomes of blood pressure self-measurement. Research showed that the elimination of the white-coat effect results in better and more accurate measurement data [20]. However, the opportunity to shift from tertiary to secondary preventive care and the implementation opportunities have not been found in previous studies. This is the first study that presents a Care Pathway for blood pressure monitoring and provides a detailed overview of the findings from this research concerning a new secondary preventive health service. In the current process, GPs are not able to filter their systems for at-risk patients and therefore secondary preventive care can be started only after the patient has been in contact with the GP and high blood pressure is detected. With the use of the Care Pathway, more data about the patient’s blood pressure is gathered and available. Both the patient and GP can review the measured data. The device has become part of the data model. It automatically transfers the data to the health record system, even when patients perform the measurement themselves. The Care Pathway design can help overcome implementation barriers [17]. The Care Pathway displays the touchpoints and role division between patients and health care professionals, which can help solve the lack of collaboration between health care parties.

The Care Pathway visualizes a clear role division and indicates that high involvement is needed to increase collaboration between the parties. Prior research found implementation barriers related to the shortage of funding [13]. To successfully implement blood pressure eHealth for patients at home, the financial responsibilities must be specified in detail. In support of this, the pathway design can now serve as a boundary object—an object with the capacity of translating and transferring between different viewpoints—to discuss the financial exchanges between the roles and organizations involved in the embedding of eHealth services. This next step in the embedding of eHealth services into the organization is also part of broader research efforts across types of eHealth devices and across countries and health care systems that focus on the implications and adaptations of the service and financial models. With the comparison of similar studies on the design of care pathways for eHealth devices at home, a next frontier in research is to develop comprehensive service models based on the data exchanges and transaction between the different parties in the networks.

### Limitations and Implications for Further Research

Despite the strengths of generative sessions with multiple stakeholders, several limitations need to be discussed. The Care Pathway created in the current study is primarily based on the Dutch health care system. For implementation in other countries, additional design research is required. To repeat the method of inquiry and reuse the visual mapping toolkit to redesign clinical workflows of blood pressure self-measurement into care pathways in other countries in Europe, the United States and Asia will add to the body of knowledge on care pathway design and the generalizability of these designs.

In design research, and in particular in generative sessions, the data obtained are rich and mainly dependent on the context. The sample size of this in-depth qualitative design research was limited to 6 participant sites, offering enough robustness for the pathway design. In this qualitative research, we used words and drawings as the main data source. Both need to be interpreted subjectively by the design researchers. The means of seeking objectivity that we used in this research were triangulation and multiple coding [38]. However, an important consideration in using the toolkit was to ensure that the facilitators remained objective and would not influence the “experts” while doing the mapping session. Furthermore, in our data analysis we came across differences between the patients in terms of the seriousness of their condition, their experiences of the role the disease has in their life, and their attitude towards monitoring. Some patients were more motivated to perform frequent measurements than others. Therefore, we suggest further specifying the at-risk patient group of the Care Pathway with additional quantitative research, such as a survey to investigate which patients benefit the most from the use of eHealth blood monitoring. A database query is also recommended.

A next avenue of research is also to organize pilot studies on to what extent lifestyle adaptations through monitoring can contribute to lowering a patient’s blood pressure. Furthermore, the Care Pathway could benefit from additional research that weighs the costs versus benefits of blood pressure self-measurement with eHealth [24] and focuses on the effects of the Care Pathway on cost reductions and efficiency. A final implication for further research concerns the comparison of similar studies on the design of care pathways for eHealth devices at home in order to further theorize on the principles, concept, and frameworks that are useful for the embedding of eHealth interventions in health care organizations.

### Practical Implications

For health professionals involved in eHealth innovation, this paper provides an example of a pathway model design that embeds eHealth technologies into an integrated service. The design method of generative sessions with the visual toolkit enabled the co-design of the example eHealth services. The pathway design method enabled the embedding of the eHealth devices into the service, providing the organization a person-centered perspective.

### Conclusion

This research resulted in a Care Pathway of blood pressure monitoring for at-risk patients as secondary preventive care. The Care Pathway was designed to guide the implementation of eHealth devices for self-measurement of blood pressure. It showcases the pathway of at-risk patients and increases their involvement in managing their blood pressure. Furthermore, the Care Pathway leads to more accurate and reliable blood pressure data about patients, which could contribute to lower use of medicines and better insight into lifestyle influences on blood pressure. The Care Pathway serves as a basis for a new service that uses eHealth in future health care.

## Abbreviations

eHealth: electronic health
GP: general practitioner
HREC: Human Research Ethics Committee
ICT: information and communication technology
